# Textural Changes by Mastication and Proper Food Texture for Patients with Oropharyngeal Dysphagia

**DOI:** 10.3390/nu12061613

**Published:** 2020-05-30

**Authors:** Koichiro Matsuo, Ichiro Fujishima

**Affiliations:** 1 Department of Dentistry and Oral-Maxillofacial Surgery, School of Medicine, Fujita Health University, Aichi 470-1192, Japan; 2 Department of Rehabilitation Medicine, Hamamatsu City Rehabilitation Hospital, Hamamatsu 433-8511, Japan; ifujishima@sis.seirei.or.jp

**Keywords:** mastication, oral function, modified diet, viscosity, Japanese Dysphagia Diet 2013

## Abstract

Bolus texture is a key factor for safe swallowing in patients with dysphagia since an improper texture may result in aspiration and/or pharyngeal residue. This article discusses swallowing bolus texture from two key aspects: the textural change of solid food by mastication and the current standardized definition of food texture in Japan. When swallowing a liquid bolus, the texture is mostly maintained from ingestion to swallow onset. For solid food, however, the food is crushed by chewing and mixed with saliva before swallowing; the texture of the ingested food is modified to an easily swallowable form at swallow onset by mastication. Understanding the mechanism of mastication and its assessment are therefore important in deciding the proper diet for dysphagic patients. As standardized criteria for classifying the texture of food and liquid are essential as well, this report also describes the Japanese Dysphagia Diet 2013 that is commonly used as the standardized index for dysphagic diets in Japan.

## 1. Introduction

The texture of the bolus is a key factor for safe swallowing in patients with dysphagia since an improper texture may cause aspiration and/or pharyngeal residue. The textural changes that occur before swallowing are completely different between a liquid and a solid food. For a liquid bolus, the texture is mostly maintained from ingestion to swallow onset. For solid food, however, the food is crushed by chewing and mixed with saliva to drastically alter the bolus texture before swallowing. Mastication and swallowing are sequential behaviors. Mastication alters oropharyngeal bolus transport and the timing of swallow initiation. As many intrinsic and extrinsic factors influence masticatory behavior, understanding the mechanisms involved in the sequence of mastication and swallowing is essential for providing proper diets to patients with dysphagia. The assessment of mastication and its associated factors can also help quantify oral function and support dysphagia rehabilitation. 

Standardized criteria for food and liquid textures are critical to plan and provide diets for dysphagic individuals. International terminology and definitions for a dysphagia diet were reported by Cicheo et al. in 2013 [1]. The International Dysphagia Diet Standardization Initiative (IDDSI) was proposed in 2016, and its framework is now recommended for implementation worldwide [2]. The international standardization of food texture and liquid consistency is, of course, important and quite useful. On the other hand, unique food cultures and eating behaviors are rooted in each country throughout the world as well. The food texture of dysphagia diets also differs among countries because of food culture and history. In Japan, the 5-stage Seirei Dysphagia Diets, or the so-called Dysphagia Diet Pyramid, were developed at the author’s (IF) institute in 1993 as the first standardized dysphagia diet categorization system in Japan [3]. Afterwards, the Japanese Dysphagia Diet 2013 (JDD2013) was developed by the Japanese Society of Dysphagia Rehabilitation (JSDR) based on the 5 category levels of the Dysphagia Diet Pyramid. The JDD2013 is now commonly used as the standardized index for dysphagic diets in Japan and will be discussed in the latter part of this article. The food form of JDD2013 can be converted to IDDSI.

## 2. Masticatory Function and Assessment of Its Components

### 2.1. Masticatory Behavior and Its Paradigmatic Model

#### 2.1.1. Process Model of Feeding

**Figure 1:**
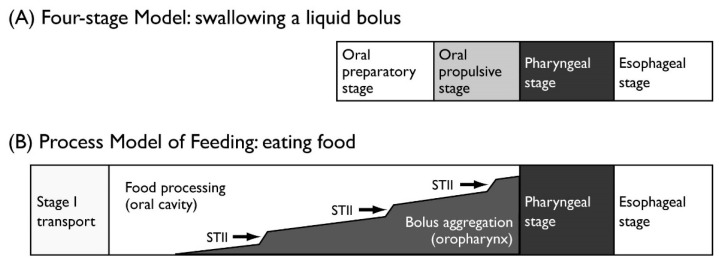
Models for consuming (**A**) liquids and (**B**) solids. (**A**) Four-stage Model. When drinking liquids, there is minimal temporal overlap between stages. (**B**) Process Model of Feeding. When eating solid food, the timing of food processing and stage II transport (STII; with aggregation in the oropharynx) can overlap substantially.

Mastication is the starting point of digestion. Chewed food is formed into a bolus and transported to the esophagus through the pharynx. The process of oral transport for solid food is different from that for a liquid bolus. Thus, two paradigmatic models are commonly used to describe the physiology of normal ingestion and swallowing: the Four-stage Model for drinking and swallowing liquid and the Process Model of Feeding for eating and swallowing solid food (Table 1). The normal swallow in humans is often described using a four-stage sequential model, while the Process Model of Feeding is employed to describe the mechanism of the eating and swallowing of solid food (Figure 1) [4]. When eating solid nutrients, the food is first transported to the occlusal surfaces of the molar teeth (stage I transport). Then, by chewing, the food is reduced in size and formed into a bolus with saliva (food processing). Chewed food is then transported to the oropharynx (stage II transport) and collects in the oropharynx until swallow onset. The fauces is open during chewing, which communicate with the oral cavity and pharynx during this period. Food processing (chewing) often continues during stage II transport and bolus aggregation in the oropharynx. Thus, when eating solid food, the stages of food processing and stage II transport can temporally overlap.

#### 2.1.2. Kinematic Linkage in Mastication

Food processing is basically conducted by masticatory jaw movement, but also involves other organs, such as the tongue, cheek, soft palate, and hyoid bone, which are coordinated with rhythmic jaw movements.

**Figure 2:**
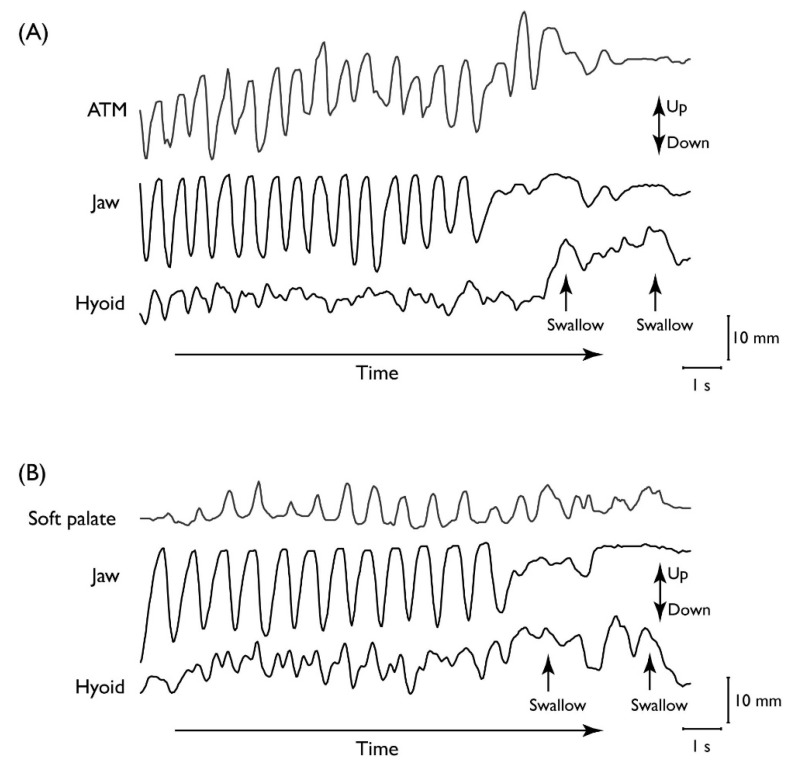
Movements of the jaw, hyoid, and (**A**) tongue or (**B**) soft palate over time. Vertical positions of (**A**) the anterior tongue marker (ATM), lower jaw, and hyoid bone, and (**B**) the soft palate, lower jaw, and hyoid bone in a complete feeding sequence. Movement towards the top of the figure is upwards. The positions of the structures are plotted relative to the upper jaw over time. Rhythmic movement of the tongue and soft palate is temporally linked to cyclic jaw movement.

During food processing, food particles are reduced in size by mastication and softened by saliva until the food texture is appropriate for swallowing. Rhythmic jaw movements for chewing continue until all of the food is prepared for swallowing, with only momentary pauses for each swallow. The cyclic movement of the jaw while processing is tightly coordinated with movements of the tongue, cheek, soft palate, and hyoid bone. During food processing, the tongue and soft palate move cyclically in association with masticatory jaw movement (Figure 2) [5,6]. This keeps the fauces open so there is no seal between the oral cavity and pharynx [7,8]. Jaw closing decreases the volume of the oral cavity and pumps air into the nasal cavity through the pharynx, delivering the food’s aroma to the chemoreceptors in the nasopharynx and nasal cavity [9]. 

Tongue movement during chewing is temporo-spatially linked with masticatory jaw movement (Figure 2A) [10,11] and is large in the anteroposterior, vertical, and lateral dimensions. The tongue generally moves forward as the jaw opens and backward as it closes. The tongue also travels superio-inferiorly and mediolaterally and rotates on its long (anteroposterior) axis during chewing [11,12]. Those movements help keep the food on the occlusal surface of the lower teeth. As the jaws are biting, the food particles on the occlusal surfaces are squeezed off. The tongue and cheeks alternately push the dropped food particles back onto the lower teeth during jaw opening. 

The soft palate also moves rhythmically during mastication in a temporal link with jaw movement (Figure 2B) [5,8]. The soft palate elevates intermittently during mastication, but the timing of this action is completely different from that during swallowing [13]. While chewing, the soft palate moves upward as the jaw opens and downward as the jaw closes. This masticatory soft palate elevation is less frequent in inspiration than in expiration, probably because soft palate elevation is suppressed to maintain retropalatal airway patency during inspiration (Figure 2B) [5]. 

#### 2.1.3. Stage II Transport

**Figure 3:**
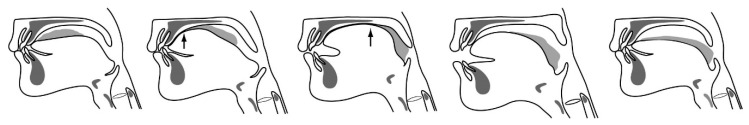
Schematic images of stage II transport. The tongue squeezes the bolus backward along the palate, through the fauces, and into the pharynx. The bolus head reaches the valleculae while food processing continues.

Chewed food is mixed with saliva and formed as a bolus on the dorsal surface of the tongue. The bolus is propelled back through the fauces to the oropharynx by a tongue squeezing motion against the palate (stage II transport; Figure 3) [4,7]. Stage II transport is primarily driven by the tongue and does not require gravity [14,15]. The mechanism for the oral propulsive phase when drinking liquids is similarly a squeeze-back mechanism. The neural mechanism underlying stage II transport is currently unknown. However, it is possible that the same neural mechanism controls the oral squeeze-back action for liquid swallows and the stage II transport of masticated solid food. The main differences between stage II transport and the oral propulsive phase for liquid drinking are that stage II transport occurs intermittently during chewing and the bolus is accumulated in the oropharynx or valleculae, while oral propulsion for liquid drinking is always followed immediately by pharyngeal swallowing. 

#### 2.1.4. Factors Influencing Masticatory Performance

Masticatory performance is basically controlled by the central nervous system, but is also influenced by such internal factors as dentition, and saliva production, as well as by external factors including the texture and other physical properties of food (Table 2). 

(1) Dentition 

Dentition has a significant impact on masticatory performance. Missing teeth, decreased occlusal contact area, use of prosthetic devices, and reduced bite force can all diminish masticatory ability [17,18,19,20]. Subjects with a decreased number of post-canine teeth or reduced functional occlusal contact area need more chewing strokes than do subjects with natural dentition [17,18]. Moreover, the particle size of the swallowed bolus is larger in individuals with decreased dentition or denture wearers due to lower masticatory efficiency [18,21]. The number of occlusal units has a significant influence on swallowing threshold, defined here as the number of chewing strokes used to process a given piece of food for swallowing [17,22]. Although there is a slight effect of gender or age on masticatory performance when eliminating the confounding effects of missing teeth or other illnesses [16], substantial variation exists in masticatory performance among individual subjects [7,17]. 

(2) Saliva Production

Saliva plays an important role in mastication. Saliva flow rates are increased by the presence of food in the mouth and are further augmented by mastication. When chewing, saliva lubricates the food to assist in bolus formation on the tongue surface before swallowing. Saliva is also important in the perception of taste and flavor. When food particles are mixed with saliva, it is easier to perceive their taste and flavor released from the food matrix [23]. 

Salivary gland hypofunction and xerostomia (dry mouth) are produced by various conditions, such as Sjögren’s syndrome and radiation therapy for cancer of the head or neck [24]. Various medications with anticholinergic side effects hamper saliva flow as well. Salivary gland hypofunction, xerostomia, and the subjective sensation of a dry mouth can lead to the deterioration of oral health and health-related quality of life. Patients with xerostomia have trouble eating due to an inability to lubricate masticated food and the resulting difficulty in forming a bolus. The perception of taste and flavor sensation can also be impaired, causing a loss in the enjoyment of eating. 

(3) Food Properties

Food properties significantly alter masticatory behavior, including food processing and stage II transport [7]. The number of chewing cycles and chewing duration both increase with the hardness of food [16,25]. The amount of time that the food is accumulated on the valleculae is also extended with greater food hardness [7]. Meanwhile, food dryness influences masticatory performance by increasing the duration of chewing cycles needed to reduce food particle size. Dry food facilitates the secretion of saliva more than does moist food.

#### 2.1.5. Factors Initiating the Pharyngeal Swallow

The location of the leading edge of the food at the time of swallow initiation is significantly different for food than for a liquid bolus. In the latter case, the bolus is held in the mouth until swallow onset, whereas the leading edge reaches the valleculae while eating solid food. Prinz and Lucas have hypothesized that the cohesiveness of the food bolus is optimized for swallowing, which depends on the size of food particles (a function of mastication) and mixture with the proper quantity of saliva [26]. Having reached optimal cohesiveness, the food particles processed by mastication and salivation are then transported to the pharynx (stage II transport) for bolus formation before swallowing. The optimized cohesive forces help to maintain bolus integrity in the pharynx both before and during swallowing, thus reducing the risk of aspiration. Prinz and Lucas have further suggested that if swallowing is delayed, excessive saliva can flood the bolus, separate the particles, reduce cohesion, and increase the risk of bolus integrity loss. This is similar to the situation that arises when eating a biphasic food that includes both soft solid and thin liquid components, such as miso soup with tofu in Japan [14,27]. As predicted by Prinz and Lucas, the low viscosity liquid component can flow rapidly down to the hypopharynx a few seconds before swallowing under the influence of gravity, while the solid component remains in the oral cavity for food processing. When liquid enters the hypopharynx during chewing, it approaches the laryngeal vestibule at a time when the larynx remains open. This may cause aspiration, especially in patients with dysphagia and impaired swallow initiation.

### 2.2. Assessment of Oral Function

#### 2.2.1. Oral Hypofunction in Older Adults

Mastication is an integrated function produced by the organs in the oral cavity and pharynx. With aging, oral function gradually deteriorates, but clinical symptoms can be easily missed due to the compensatory mechanisms among organs. In order to preserve and improve oral health, the quantitative assessment of declines in specific oral functions and the early diagnosis of hypofunction are important. 

The concept of “oral hypofunction” (OHF) was proposed by the Japanese Society of Gerodontology to describe the deterioration of oral function and call attention to oral function decline in older adults [28]. Rather than originating from a single source, OHF is regarded as a complex pathophysiological condition caused by the combined deterioration of several oral functions. OHF comprises seven sub-symptoms and is diagnosed if at least three symptoms meet cut-off criteria (Table 3). By measuring OHF quantitatively, both the examiner and patient can understand the patient’s oral status to enable prompt intervention in the early stages of oral function deterioration, thus preventing further impairments in eating function. In dysphagia rehabilitation, the quantitative measurement of individual oral functions can also be used as indicators of improvement in masticatory and eating function. 

#### 2.2.2. Assessment of Oral Function

**Figure 4:**
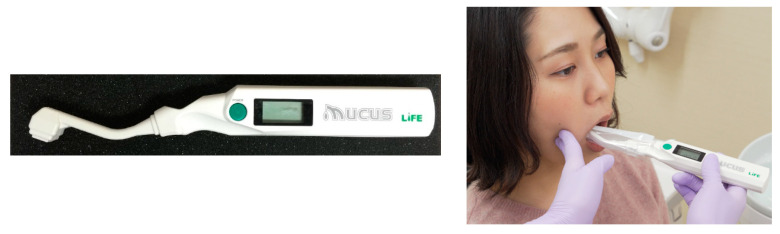
Oral moisture checker.

**Figure 5:**
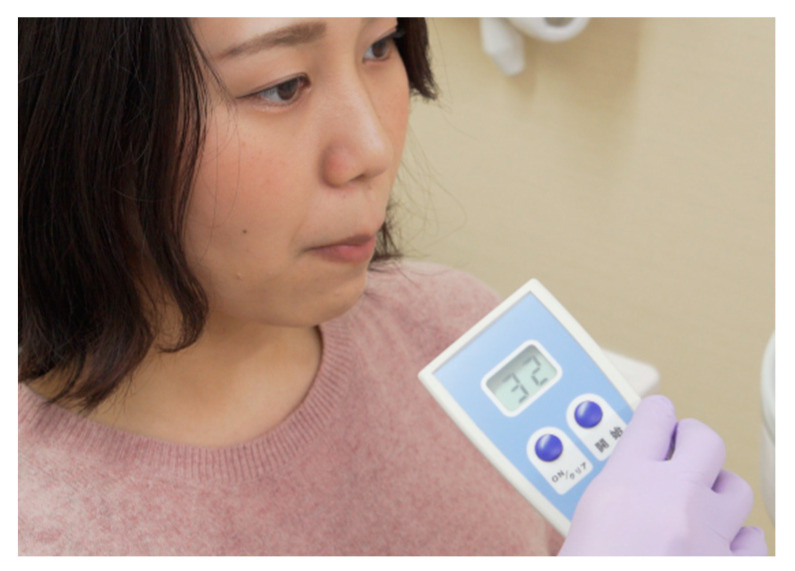
Digital counter.

**Figure 6:**
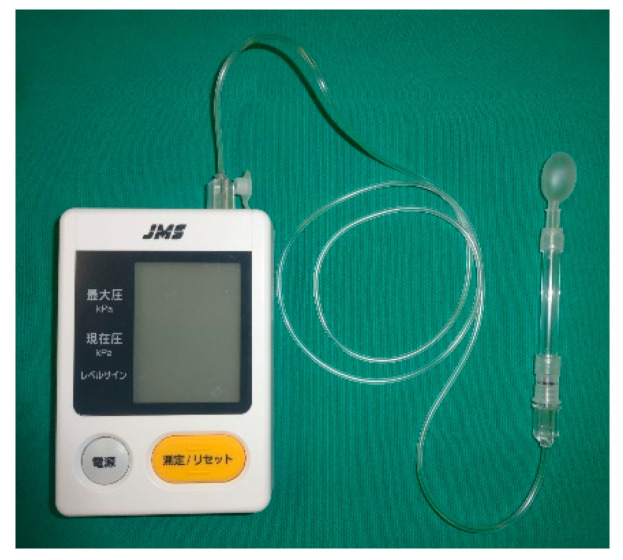
Digital tongue pressure meter.

**Figure 7:**
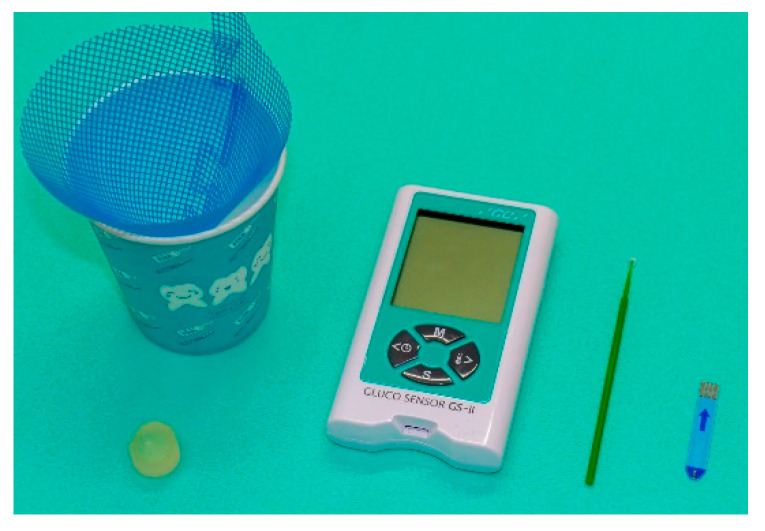
Masticatory ability testing system.

Each oral function parameter is measured with specialized instruments and scored quantitatively. The details of measurements and OHF cut-off thresholds are described in a previous report [28], as follows: 

(1) Oral hygiene: The amount of bacteria on the dorsal tongue surface is measured by a bacteria detection apparatus (Bacteria counter, Panasonic Healthcare, Tokyo, Japan), and bacteria counts are evaluated as indicators of oral hygiene. A sterilized swab is swiped three times in a 10 mm swath on the middle of the dorsal tongue surface and then placed in distilled water in the bacteria detection apparatus [29]. 

(2) Oral wetness: The wetness of the buccal mucosal surface is assessed using an oral moisture checker (Mucus, Life Co., Ltd., Japan) (Figure 4) [30]. The sensor of the instrument is attached to the right-side buccal surface of the participant for 2 s, and the degree of oral wetness is measured in triplicate at the same site for the calculation of mean values. 

(3) Occlusal force: Occlusal force is measured by 3 s of clenching using pressure-indicating film (Dental prescale sheet, GC, Japan) [31]. The area of changed color on the sheet by clenching is evaluated by analysis software to calculate occlusal force. 

(4) Tongue–lip motor function: Participants are instructed to say the syllables /pa/, /ta/, or /ka/ as many times as possible within 5 s. The number of utterances (/sec) is counted by a digital counter (Kenkokun Handy, Takei Scientific Instruments Co., Ltd., Japan) (Figure 5) [32]. 

(5) Tongue pressure: A tongue pressure sensor balloon probe connected to a digital tongue pressure meter (JMS, Hiroshima, Japan) (Figure 6) is placed on the dorsal tongue surface. Participants are asked to press up against the probe with their tongue towards the hard palate at maximum strength for 3 s [33]. 

(6) Masticatory function: Masticatory function is measured using a gummy jelly. Participants are instructed to chew 2 g of gummy jelly without swallowing the bolus or saliva for 20 s. They are then asked to hold 10 mL of distilled water in their mouth and spit out the jelly and water into a cup fitted with a funneled mesh. The amount of eluted glucose is measured with a masticatory ability testing system (Gluco Sensor GS-II, GC, Japan) (Figure 7) [34]. 

(7) Swallowing function: Swallowing function is assessed by a self-administered questionnaire for swallowing (EAT-10) and expressed as a numerical score from 0 to 40 [35]. 

## 3. Proper Modification of Food Texture for Oropharyngeal Dysphagia

### 3.1. Standardized Texture Levels

**Figure 8:**
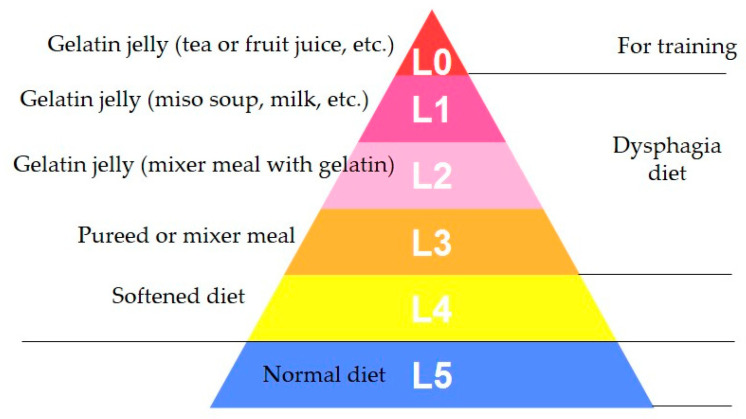
Schema of 5-stage Seirei Dysphagia Diets (Dysphagia Diet Pyramid).

Dysphagia rehabilitation for stroke at Seirei Mikatahara General Hospital was started in 1989. When the dysphagia team realized the importance of dysphagia diets, 5-stage Seirei Dysphagia Diets were developed using sensory evaluations, videofluoroscopic examination of swallowing (VF) findings, and clinical observations. These diets were published in 1993 [3], and later named as the “Dysphagia Diet Pyramid” by a team member, dietitian Ms. Setsuko Kanaya, in 2004 (personal communication) (Figure 8). 

The 5-stage Seirei Dysphagia Diets (Dysphagia Diet Pyramid) consist of five categories from L0 to L4: L0 is gelatin jelly (tea or fruit juice, etc.), L1 is gelatin jelly (miso soup, milk, etc., containing protein), L2 is gelatin jelly (mixer meal with gelatin), L3 is a puréed or mixer meal, and L4 is a softened diet. Gelatin jelly was mainly used in the first stage of swallowing training due to no better alternative at the time.

We evaluated the frequency of aspiration/penetration and pharyngeal residues among the different types of food of the 5-stage Seirei Dysphagia Diets using VF [36]. A total of 121 patients with dysphagia (85 male and 36 female; average age: 72.7 ± 10.0 years) underwent VF. The cause of dysphagia was cerebral infarction in 46 patients (37%), aspiration pneumonia in 18 patients (15%), cerebral hemorrhage in 13 patients (11%), neuromuscular disease in 13 patients (11%), gastrointestinal disease in 8 patients (7%), and other in 23 patients (19%). The severity of dysphagia was determined by using the Food Intake Level Scale [37]. VF was performed for six different types of food containing 40% barium sulfate: sliced and crushed gelatin, mildly and extremely thickened water, crushed agar jelly, and water. A xanthan gum-based thickener was used for thickening the water. The χ2 test and Fisher’s exact test were employed for statistical analysis. The significance level was corrected by Bonferroni correction for multiple comparisons, and p < 0.0033 was considered statistically significant.

**Figure 9:**
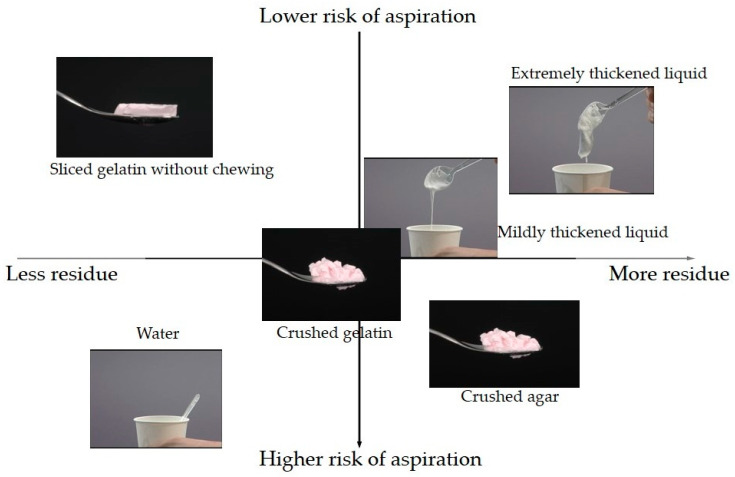
Tendencies of aspiration and pharyngeal residue among food types.

Penetration/aspiration was markedly more frequent for thin water than for all other types of food (Table 4) and was less frequent for sliced gelatin jelly without chewing than for crushed gelatin or agar jellies. Penetration/aspiration was also less frequent for extremely thickened water than for crushed gelatin, agar jellies, or mildly thickened water. Pharyngeal residue was remarkably lower for sliced gelatin and water than for mildly or extremely thickened water or crushed agar jelly (Table 5). These results suggested that sliced gelatin jelly was the safest type of food for dysphagia patients, followed next by extremely thickened water. Based on these findings, the Dysphagia Diet Pyramid was judged as valid and has since been widely adopted in Japan. Figure 9 shows the tendency of aspiration and pharyngeal residue among different types of food.

Although the Dysphagia Diet Pyramid became widely known in Japan, several other kinds of dysphagia diets existed at various institutions nationwide. To avoid confusion, a standardized dysphagia diet system was needed for patients, their families, and medical staff in the patient care network. Especially, a uniform name was required. We organized a special committee for a modified dysphagia diet system in the JSDR in 2010 to categorize the levels of modified foods and thickened liquids. The special committee developed “The tentative draft of 5-stage modified dysphagia diet” in 2011 and “The standard draft of Japanese Dysphagia Diet 2012” in 2012 and published them on paper and on the website. Public and professional feedback culminated in the JDD2013 consisting of a precise explanation text and tables for food and liquid [38].

**Figure 10:**
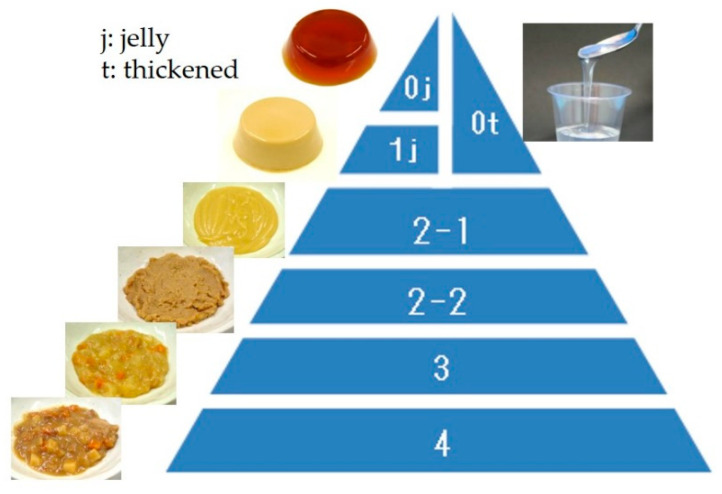
Schema of JDD2013.

The JDD2013 consists of five categories (Figure 10). Code 0j is swallow-training jelly, which can be placed directly in the mouth and swallowed without chewing (i.e., can be swallowed whole). Considering infection and tissue reactions to pulmonary aspiration, protein content is kept low. Code 0t is thickened liquid, which applies to tea or fruit juice thickened with a thickening-adjustment food. If the food contains protein, it is considered Code 1j. Code 1j is a jelly/pudding/mousse-like food product that does not require abilities related to chewing. It is homogeneous and soft, with little syneresis. Code 2 is food in the form of a paste that is sub-classified into 2-1 or 2-2 based on heterogeneity. Code 2-1 is food with smooth and homogeneous materials under 850 μm, while Code 2-2 is for inhomogeneous materials containing soft grains (i.e., a blender diet). Typically, Code 2 foods are considered blender, mixer, puréed, or paste foods. Code 3 food contains solid forms, which can be crushed even without teeth into a bolus. Code 4 food can be handled with teeth and is difficult to crush between the tongue and palate. Some general foods of not too hard, not too sticky, and not too crumbly are classified into this category. JDD2013 did not determine the physical property evaluation so that it can be used by general clinicians. However, since JDD2013 and Dysphagia Diet Pyramid are compatible [38], measured variables in the Dysphagia Diet Pyramid are shown in Table 6 as the references [39,40].

**Figure 11:**
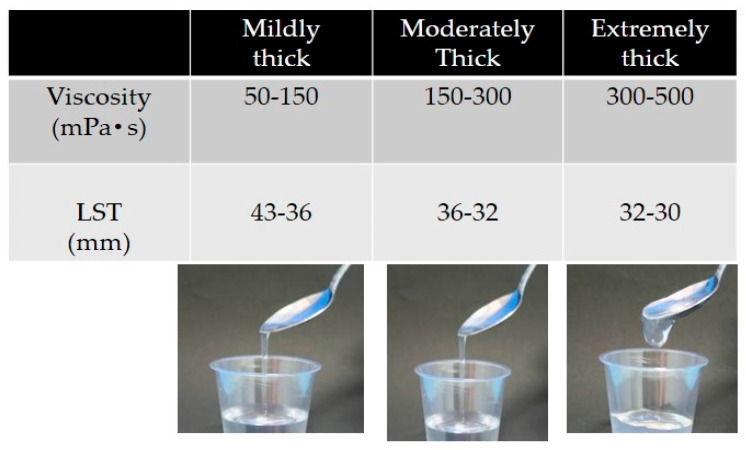
Criteria of thickened liquids (Viscosity obtained share rate 50 sec^−^) LST: line spread test.

Liquids are thickened by a xanthan gum-based thickener and are divided into three categories: mildly, moderately, and extremely thick (Figure 11) [41]. Mildly thick liquids flow quickly by tilting the spoon and leave a thin trace of residue in the cup after poured. They are suitable for patients with mild dysphagia. Moderately thick liquids flow slowly by tilting the spoon and leave a film of residue in the cup after being poured. They are appropriate for patients with moderately severe dysphagia. Extremely thick liquids maintain most of their form after tilting the spoon and flow slowly while being poured. They are suitable for patients with more severe dysphagia who display a risk of aspiration using moderately thick liquids. Viscosity levels are 50–150 mPa·s for mildly thick, 150–300 mPa·s for moderately thick, and 300–500 mPa·s for extremely thick liquids, with a Viscometer share rate of 50 sec^−1^.

### 3.2. Assessment of Proper Texture for Patients with Oral Dysphagia

**Figure 12:**
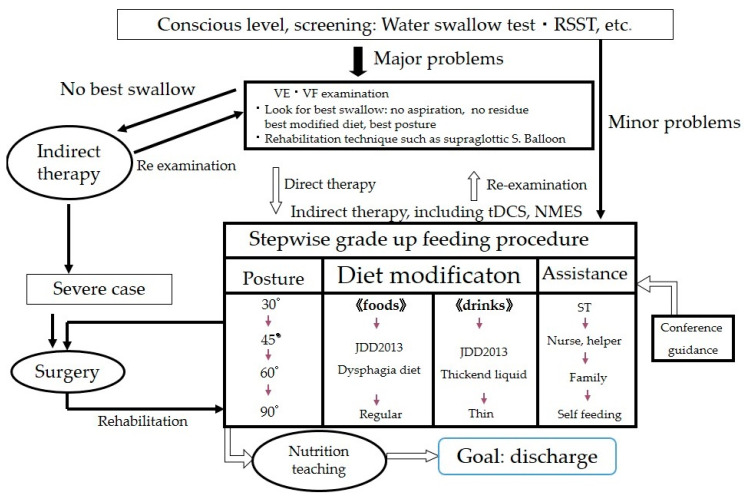
Flow chart of dysphagia rehabilitation. Balloon: Balloon dilatation of Upper Esophageal Sphincter, tDCS: transcranial Direct Current Stimulation, NMES: neuromuscular electrical stimulation.

When starting dysphagia therapy, two critical conditions require verification: patient’s conscious level is clear and his or her general condition is stable (i.e., normal vital signs and respiration, no cough or excess sputum). The oral cavity should first be cleaned. For screening, we use the 3 mL and/or 30 mL water swallow test with/without liquid thickener and the Repetitive Saliva Swallow Test (RSST) (three times or more is normal). If there are no problems, we commence meals with a normal diet. If a problem is found, we begin a dysphagia diet with close observation. Instrumental examination is performed for difficult cases. Our basis for applying direct therapy is safe swallowing and errorless training, as depicted in Figure 12.

**Figure 13:**
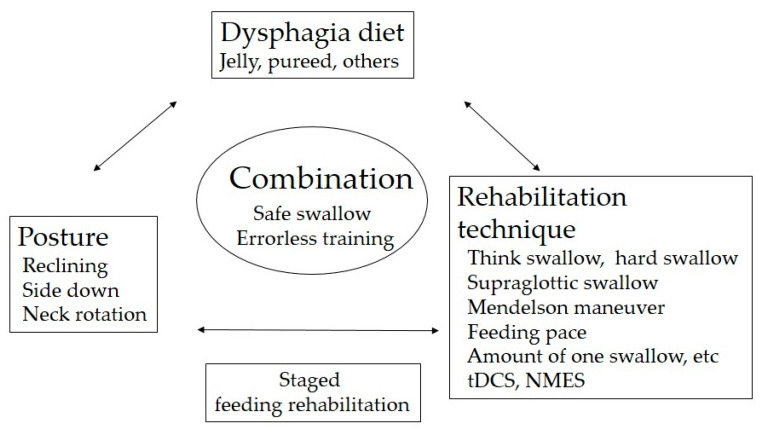
The proper combination of food, posture, and rehabilitation technique is important for safe swallowing and errorless training.

The key to successful dysphagia rehabilitation is the choice of proper food and body position and the use of rehabilitation techniques to enable no aspiration and residue-free eating training. The combination of these 3 elements is important (Figure 13). We use VF and/or videoendscopic examination of swallowing (VE) to select the most suitable food for the patient’s swallowing function status, and conduct direct swallowing training in an easy-to-eat posture using rehabilitation techniques. Such step-by-step safe feeding training leads to improvements in swallowing function. 

Food texture is essential in dysphagia rehabilitation. We discussed swallowing bolus texture from two key aspects: the textural change of solid food by mastication and the current standardized definition of food texture in Japan. Since mastication changes the texture of ingested food dramatically, understanding masticatory mechanism and its influencing factors is important to provide dysphagia diet. As standardized criteria for classifying the texture of food and liquid are essential as well, we introduced the Japanese Dysphagia Diet 2013 that is commonly used as the standardized index for dysphagia diets in Japan.

## Figures and Tables

**Table 1:** Description of stages in the Four-stage Model and Process Model of Feeding.

	Stage	Description
**Four-stage Model for liquids**		
	Oral preparatory stage	A liquid bolus is held in the mouth.
	Oral propulsive stage	The bolus is propelled to the pharynx.
	Pharyngeal stage	The bolus is transited to the esophagus through the UES.
	Esophageal stage	The bolus is transported in the esophagus by peristalsis.
**Process Model of Feeding for solid food**		
	Stage I transport	Ingested food is transported to the molar region.
	Processing	The food is reduced in size and a bolus is formed with saliva.
	Stage II transport	The formed bolus is transported to the oropharynx and collected until swallow onset.
	Swallowing	The bolus is transited to the esophagus through the UES.

UES, upper esophageal sphincter.

**Table 2:** Internal and external factors affecting masticatory function [16].

Influence Factor	Effect on Masticatory Function
**Internal factors**	
Loss of post-canine teeth,	Increased number of chewing strokes.
reduced area of functional	Larger particle size of the swallowed bolus.
occlusal contact, or	
use of dentures	
Age or gender	Little effect on masticatory performance.
**External factors**	
Hardness of food	Increased number of chewing strokes.
	Longer duration of chewing and pharyngeal aggregation.
Dryness of food	Increased chewing duration, more saliva.

**Table 3:** Seven oral sub-symptoms of OHF and their criteria [28].

Oral Sub-Symptom	Cut-Off Criterion
Oral hygiene	Total number of bacteria > 10^6.5^ CFU/mL.
Oral dryness	Measured value with a moisture checker < 27.0.
Occlusal force	Occlusal force < 200 N.
Tongue-lip motor function	Utterance count of /pa/, /ta/, or /ka/ < 6/s.
Tongue pressure	Maximum tongue pressure < 30 kPa.
Masticatory function	Glucose concentration in chewing test < 100 mg/dL.
Swallowing function	Total Eating Assessment Tool (EAT-10) score ≥ 3.

**Table 4:** Differences in the frequency of aspiration or penetration among food types.

Object 1	Object 2	N	*p*-Value	Frequent Object
Sliced gelatin without chewing	Crushed gelatin	37	0.000 **	Crushed gelatin
	Extremely thickened liquid	85	0.201	
	Mildly thickened liquid	69	0.700	
	Water	39	0.000 **	Water
	Crushed agar	63	0.019 *	Crushed agar
Crushed gelatin	Extremely thickened liquid	62	0.004 *	Crushed gelatin
	Mildly thickened liquid	54	0.267	
	Water	33	0.001 **	Water
	Crushed agar	48	0.805	
Extremely thickened liquid	Mildly thickened liquid	89	0.008 *	Mildly thickened liquid
	Water	52	0.000 **	Water
	Crushed agar	80	0.001 **	Crushed agar
Mildly thickened liquid	Water	51	0.000 **	Water
	Crushed agar	77	0.138	
Water	Crushed agar	50	0.000 **	Water

* *p* < 0.05; ** *p* < 0.0033.

**Table 5:** Differences in the frequency of pharyngeal residue among food types.

Object 1	Object 2	N	*p*-Value	Less Object
Sliced gelatin without chewing	Crushed gelatin	37	0.104	
	Extremely thickened liquid	85	0.002 **	Sliced gelatin without chewing
	Mildly thickened liquid	69	0.011 *	Sliced gelatin without chewing
	Water	39	0.631	
	Crushed agar	63	0.000 **	Sliced gelatin without chewing
Crushed gelatin	Extremely thickened liquid	62	0.850	
	Mildly thickened liquid	54	0.850	
	Water	33	0.044 *	Water
	Crushed agar	48	0.157	
Extremely thickened liquid	Mildly thickened liquid	89	0.758	
	Water	52	0.002 **	Water
	Crushed agar	80	0.050	
Mildly thickened liquid	Water	51	0.017 *	Water
	Crushed agar	77	0.021 *	Mildly thickened liquid
Water	Crushed agar	50	0.000 **	Water

* *p* < 0.05; ** *p* < 0.0033.

**Table 6:** The Texture Profile Analysis (adhesiveness, cohesiveness and hardness) of the Dysphagia Diet Pyramid and the JDD2013.

Dysphagia Pyramid: Level	0	1,2	3	4
JDD2013: Code	0j	1j	2-1, 2-2	3, 4
Hardness (N/m2)	2000–7000	0–12,000	≤15,000	≤40,000
Cohesiveness	0.2–0.5	0.2–0.7	0.2–0.9	0–1.0
Adhesiveness (J/m3)	≤200	≤300	≤1000	≤1000
